# Supplementary material for: Relationship between accelerometer-measured sleep duration and Stroop performance: a functional near-infrared spectroscopy study among young adults
Source: PeerJ. 2024 Feb 28;12:e17057. doi: 10.7717/peerj.17057 (PMC10908256; doi:10.7717/peerj.17057)
Supplement: Supplemental Information 3 [file peerj-12-17057-s003.docx]

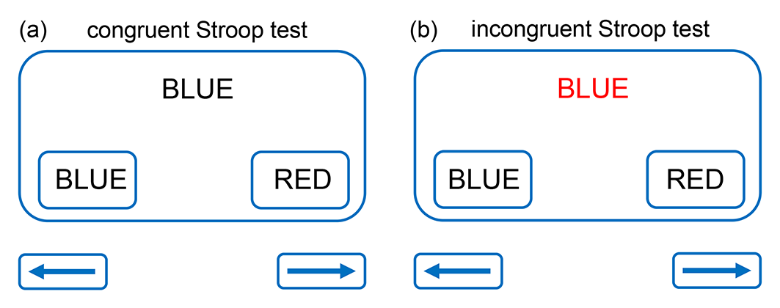


Figure S1. Examples of the congruent and incongruent Stroop tests in this study.


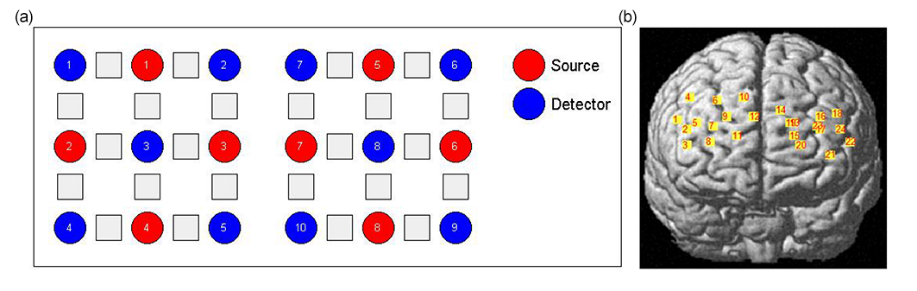


Figure S2. (a) Probe set of sources and detectors on the prefrontal cortex by 2D visualization; (b) fNIRS probe set and red numbers with yellow background represents for the position of the 24 channels by 3D visualization.

**Table S1**. The MNI coordinates and anatomical labels corresponding to the 24 measurement channels.

|  | MNI | | | **Brain Regions (Percentage of Overlap)** |
| --- | --- | --- | --- | --- |
|  | x | *y* | z |  |
| Channel 1 | 52 | 38 | 28 | Middle Frontal Gyri (0.60) |
| Channel 2 | 46 | 51 | 22 | Middle Frontal Gyri (1) |
| Channel 3 | 47 | 54 | 13 | Middle Frontal Gyri (1) |
| Channel 4 | 44 | 36 | 42 | Middle Frontal Gyri (1) |
| Channel 5 | 40 | 54 | 26 | Middle Frontal Gyri (1) |
| Channel 6 | 27 | 52 | 40 | Dorsolateral Superior Frontal Gyri (0.61) |
| Channel 7 | 31 | 63 | 23 | Middle Frontal Gyri (0.57) |
| Channel 8 | 32 | 66 | 15 | Dorsolateral Superior Frontal Gyri (0.75) |
| Channel 9 | 22 | 63 | 30 | Dorsolateral Superior Frontal Gyri (0.65) |
| Channel 10 | 13 | 57 | 42 | Superior and Middle Frontal Gyri (0.56) |
| Channel 11 | 16 | 71 | 19 | Dorsolateral Superior Frontal Gyri (0.53) |
| Channel 12 | 6 | 65 | 31 | Superior and Middle Frontal Gyri (0.58) |
| Channel 13 | -17 | 66 | 26 | Dorsolateral Superior Frontal Gyri (0.84) |
| Channel 14 | -9 | 64 | 33 | Superior and Middle Frontal Gyri (0.62) |
| Channel 15 | -17 | 70 | 18 | Dorsolateral Superior Frontal Gyri (0.95) |
| Channel 16 | -33 | 54 | 30 | Middle Frontal Gyri (0.89) |
| Channel 17 | -35 | 59 | 23 | Middle Frontal Gyri (0.80) |
| Channel 18 | -45 | 41 | 32 | Middle Frontal Gyri (0.88) |
| Channel 19 | -16 | 66 | 26 | Dorsolateral Superior Frontal Gyri (0.75) |
| Channel 20 | -23 | 70 | 13 | Dorsolateral Superior Frontal Gyri (0.98) |
| Channel 21 | -41 | 59 | 6 | Middle Frontal Gyri (0.75) |
| Channel 22 | -52 | 41 | 14 | Superior and Middle Frontal Gyri (0.81) |
| Channel 23 | -31 | 60 | 24 | Middle Frontal Gyri (0.63) |
| Channel 24 | -46 | 47 | 21 | Middle Frontal Gyri (0.79) |

**Table S2**. The associations between sleep duration with the congruent and incongruent Stroop test reaction time.

|  | Reaction time of congruent Stroop test | | Reaction time of incongruent Stroop test | |
| --- | --- | --- | --- | --- |
|  | β (95%CI) | *P-value* | β (95%CI) | *P-value* |
| **Sleep duration as continuous variable** |  |  |  |  |
| Sleep (min/day) | 0(-0.003,0.003) | 0.955 | -0.001(-0.005,0.004) | 0.899 |
| **Sleep duration as category variable** |  |  |  |  |
| Regular sleep duration | Reference |  | Reference |  |
| Light short sleep | 0.026(-0.027,0.079) | 0.326 | 0.025(-0.043,0.093) | 0.467 |
| Mild short sleep | 0.008(-0.040,0.055) | 0.752 | 0.013(-0.048,0.074) | 0.674 |
| Severe short sleep | -0.003(-0.049,0.043) | 0.900 | 0.014(-0.046,0.073) | 0.648 |

**Table S3.** The associations between the regional cortical changes in the concentrations of oxygenated hemoglobin (oxyHb) and accuracy of the incongruent Stroop test in the regular sleep duration group.

|  | β | Lower CI | Upper CI | *P-value* |
| --- | --- | --- | --- | --- |
| Channel 1 | -0.003 | -0.019 | 0.012 | 0.645 |
| Channel 2 | 0.001 | -0.015 | 0.017 | 0.886 |
| Channel 3 | 0.002 | -0.016 | 0.02 | 0.784 |
| Channel 4 | 0.004 | -0.004 | 0.012 | 0.32 |
| Channel 5 | 0 | -0.01 | 0.009 | 0.928 |
| Channel 6 | 0.003 | -0.018 | 0.023 | 0.773 |
| Channel 7 | 0.002 | -0.009 | 0.013 | 0.664 |
| Channel 8 | 0.001 | -0.015 | 0.017 | 0.838 |
| Channel 9 | -0.001 | -0.016 | 0.014 | 0.904 |
| Channel 10 | 0.002 | -0.01 | 0.014 | 0.695 |
| Channel 11 | -0.002 | -0.012 | 0.008 | 0.714 |
| Channel 12 | 0.003 | -0.019 | 0.024 | 0.754 |
| Channel 13 | -0.002 | -0.006 | 0.002 | 0.317 |
| Channel 14 | -0.001 | -0.005 | 0.003 | 0.693 |
| Channel 15 | 0.006 | -0.006 | 0.018 | 0.275 |
| Channel 16 | 0.007 | -0.009 | 0.023 | 0.349 |
| Channel 17 | **-0.009** | **-0.018** | **0.001** | **0.063*** |
| Channel 18 | 0.008 | -0.008 | 0.024 | 0.271 |
| Channel 19 | -0.002 | -0.009 | 0.006 | 0.596 |
| Channel 20 | 0.002 | -0.005 | 0.01 | 0.502 |
| Channel 21 | 0.008 | -0.03 | 0.046 | 0.63 |
| Channel 22 | 0.007 | -0.009 | 0.024 | 0.338 |
| Channel 23 | -0.006 | -0.017 | 0.005 | 0.229 |
| Channel 24 | 0.009 | -0.008 | 0.026 | 0.256 |

Notes: CI = confidence intervals, * Significant with p < 0.05.

**Table S4.** The associations between the regional cortical changes in the concentrations of oxygenated hemoglobin (oxyHb) and accuracy of the incongruent Stroop test in the light short sleep group.

|  | β | Lower CI | Upper CI | *P-value* |
| --- | --- | --- | --- | --- |
| Channel 1 | -0.004 | -0.013 | 0.005 | 0.334 |
| Channel 2 | 0.013 | -0.012 | 0.038 | 0.248 |
| Channel 3 | 0.013 | -0.017 | 0.043 | 0.344 |
| Channel 4 | -0.004 | -0.012 | 0.003 | 0.188 |
| Channel 5 | -0.003 | -0.021 | 0.015 | 0.716 |
| Channel 6 | 0.017 | -0.015 | 0.05 | 0.249 |
| Channel 7 | 0.012 | -0.036 | 0.059 | 0.583 |
| Channel 8 | 0.022 | -0.004 | 0.048 | 0.086 |
| Channel 9 | -0.011 | -0.193 | 0.172 | 0.894 |
| Channel 10 | -0.006 | -0.019 | 0.007 | 0.299 |
| Channel 11 | 0.003 | -0.021 | 0.026 | 0.786 |
| Channel 12 | 0.015 | -0.021 | 0.05 | 0.368 |
| Channel 13 | -0.008 | -0.022 | 0.005 | 0.19 |
| Channel 14 | 0.006 | -0.026 | 0.038 | 0.666 |
| Channel 15 | 0.012 | -0.078 | 0.103 | 0.761 |
| Channel 16 | 0.033 | -0.019 | 0.085 | 0.176 |
| Channel 17 | 0.013 | -0.02 | 0.045 | 0.387 |
| Channel 18 | -0.043 | -0.16 | 0.075 | 0.419 |
| Channel 19 | **0.024** | **0.004** | **0.045** | **0.026*** |
| Channel 20 | **0.023** | **0.007** | **0.039** | **0.011*** |
| Channel 21 | -0.03 | -0.11 | 0.05 | 0.403 |
| Channel 22 | 0.007 | -0.033 | 0.047 | 0.692 |
| Channel 23 | 0.001 | -0.019 | 0.022 | 0.877 |
| Channel 24 | 0.037 | -0.096 | 0.17 | 0.532 |

Notes: CI = confidence intervals, * Significant with p < 0.05.

**Table S5.** The associations between the regional cortical changes in the concentrations of oxygenated hemoglobin (oxyHb) and accuracy of the incongruent Stroop test in the mild short sleep group.

|  | β | Lower CI | Upper CI | *P-value* |
| --- | --- | --- | --- | --- |
| Channel 1 | 0.006 | -0.006 | 0.018 | 0.286 |
| Channel 2 | -0.005 | -0.038 | 0.027 | 0.734 |
| Channel 3 | 0.002 | -0.027 | 0.031 | 0.885 |
| Channel 4 | -0.001 | -0.008 | 0.006 | 0.769 |
| Channel 5 | 0.006 | -0.012 | 0.024 | 0.482 |
| Channel 6 | -0.015 | -0.052 | 0.023 | 0.413 |
| Channel 7 | 0.008 | -0.005 | 0.021 | 0.204 |
| Channel 8 | 0.003 | -0.01 | 0.017 | 0.605 |
| Channel 9 | -0.007 | -0.023 | 0.009 | 0.374 |
| Channel 10 | 0.004 | -0.015 | 0.024 | 0.652 |
| Channel 11 | -0.012 | -0.036 | 0.012 | 0.315 |
| Channel 12 | 0.029 | -0.03 | 0.089 | 0.308 |
| Channel 13 | 0.007 | -0.005 | 0.019 | 0.231 |
| Channel 14 | 0.003 | -0.01 | 0.016 | 0.621 |
| Channel 15 | -0.027 | -0.074 | 0.02 | 0.233 |
| Channel 16 | 0.002 | -0.015 | 0.02 | 0.765 |
| Channel 17 | -0.008 | -0.029 | 0.013 | 0.428 |
| Channel 18 | -0.006 | -0.036 | 0.025 | 0.7 |
| Channel 19 | -0.003 | -0.01 | 0.004 | 0.31 |
| Channel 20 | **-0.012** | **-0.024** | **0.001** | **0.068** |
| Channel 21 | 0.012 | -0.063 | 0.088 | 0.734 |
| Channel 22 | 0.006 | -0.01 | 0.021 | 0.44 |
| Channel 23 | 0.003 | -0.008 | 0.013 | 0.584 |
| Channel 24 | -0.008 | -0.037 | 0.02 | 0.545 |

Notes: CI = confidence intervals, * Significant with p < 0.05.

**Table S6.** The associations between the regional cortical changes in the concentrations of oxygenated hemoglobin (oxyHb) and accuracy of the incongruent Stroop test in the severe short sleep group.

|  | β | Lower CI | Upper CI | *P-value* |
| --- | --- | --- | --- | --- |
| Channel 1 | 0.003 | -0.01 | 0.016 | 0.675 |
| Channel 2 | -0.007 | -0.022 | 0.007 | 0.283 |
| Channel 3 | 0.017 | -0.016 | 0.05 | 0.295 |
| Channel 4 | 0.005 | -0.004 | 0.013 | 0.249 |
| Channel 5 | **0.014** | **0.005** | **0.023** | **0.005** |
| Channel 6 | -0.003 | -0.046 | 0.04 | 0.882 |
| Channel 7 | -0.01 | -0.029 | 0.008 | 0.264 |
| Channel 8 | **-0.017** | **-0.03** | **-0.004** | **0.016** |
| Channel 9 | 0.02 | -0.013 | 0.054 | 0.209 |
| Channel 10 | 0 | -0.013 | 0.013 | 0.996 |
| Channel 11 | **-0.014** | **-0.027** | **-0.001** | **0.035** |
| Channel 12 | **0.05** | **0.013** | **0.088** | **0.012** |
| Channel 13 | 0.006 | -0.006 | 0.017 | 0.307 |
| Channel 14 | 0.014 | 0 | 0.029 | 0.052 |
| Channel 15 | 0.029 | -0.062 | 0.12 | 0.511 |
| Channel 16 | -0.012 | -0.03 | 0.007 | 0.213 |
| Channel 17 | **-0.012** | **-0.022** | **-0.003** | **0.01** |
| Channel 18 | 0.015 | -0.041 | 0.07 | 0.576 |
| Channel 19 | 0.009 | -0.001 | 0.019 | 0.07 |
| Channel 20 | 0.003 | -0.008 | 0.014 | 0.595 |
| Channel 21 | -0.003 | -0.023 | 0.017 | 0.73 |
| Channel 22 | 0.006 | -0.01 | 0.021 | 0.432 |
| Channel 23 | -0.004 | -0.026 | 0.017 | 0.669 |
| Channel 24 | -0.013 | -0.063 | 0.036 | 0.577 |

Notes: CI = confidence intervals, * Significant with p < 0.05
